# Frequency and Direction-Dependent Shear-Wave Responses in Ex Vivo Tissues Measured by a Time-of-Flight Device

**DOI:** 10.3390/bioengineering13090959

**Published:** 2026-08-23

**Authors:** Jotham Josephat Kimondo, Ziang Feng, Jie Yang, Qiang Lu, Sandra Pérez-Buitrago, Zhe Wu

**Affiliations:** 1 School of Life Sciences and Technology, University of Electronic Science and Technology of China, Chengdu 610054, China; 2 Department of Medical Ultrasound, West China Hospital of Sichuan University, Chengdu 610041, Chinaluqiang@scu.edu.cn (Q.L.); 3 Department of Engineering, Medical Device Research Group, Pontifical Catholic University of Peru, San Miguel 15088, Lima, Peru; sm.perez@pucp.edu.pe; 4 Tianfu Jincheng Laboratory, City of Future Medicine, Chengdu 641400, China

**Keywords:** shear-wave elastography, time of flight, ex vivo tissue characterization, Kelvin–Voigt fractional derivative model, direction-dependent shear-wave response, onset detection

## Abstract

Shear-wave time-of-flight (TOF) measurement enables controlled assessment of frequency-dependent wave propagation, but its feasibility in biological tissues remains insufficiently established. This study evaluated whether a custom shear-wave TOF device could detect frequency- and direction-dependent responses in ex vivo tissues. Three porcine liver samples and three chicken breast samples were examined. Chicken breast was measured with propagation parallel and perpendicular to visible muscle fibers. One-cycle sinusoidal excitations were applied at 40–160 Hz, with 50 acquisitions ensemble-averaged per sample–frequency measurement. TOF was estimated using Tx threshold detection and cumulative-energy-based Rx onset detection, and TOF-derived apparent shear-wave propagation speed was calculated from the Tx–Rx distance and the measured TOF. Frequency-dependent data were fitted using the Kelvin–Voigt fractional derivative model to obtain model-dependent KVFD fit parameters. Signal quality was assessed, and a preliminary descriptive comparison with HISKY EQTouch UD3000 (Wuxi Hisky Medical Technologies Co., Ltd., Wuxi, China) SWE was performed. All 63 averaged sample–frequency measurements satisfied the predefined primary-detection criteria. Mean apparent shear-wave speed was 3.145 m/s in porcine liver, 6.133 m/s in chicken breast measured parallel to the fibers, and 5.914 m/s in chicken breast measured perpendicular to the fibers, giving a parallel-to-perpendicular speed ratio of 1.037. Mean post-averaging, post-processing SNR ranged from 24.47 to 31.52 dB. The UD3000 comparison showed the same tissue ranking. The device detected frequency- and direction-dependent responses in averaged ex vivo signals, supporting its feasibility as a controlled research platform. Claims of absolute stiffness accuracy and intrinsic muscle anisotropy require independent calibration and validation.

## 1. Introduction

Quantitative assessment of soft-tissue mechanical behavior has become an important component of biomedical imaging. Changes in stiffness and viscoelastic response are closely associated with tissue structure, composition, and disease-related remodeling [1,2]. Ultrasound elastography has been widely developed for this purpose because it enables non-invasive estimation of tissue stiffness by measuring the tissue response to an externally or acoustically induced mechanical excitation [3]. Among elastography techniques, shear-wave-based methods are particularly useful because shear-wave speed is directly related to tissue stiffness under appropriate mechanical assumptions, enabling quantitative mechanical assessment beyond qualitative palpation-like imaging [3,4,5].

Modern shear-wave elastography (SWE) systems commonly generate a transient mechanical disturbance and estimate shear-wave speed from the measured wave propagation through tissue [2,6]. Supersonic shear imaging and related shear-wave elastography approaches have demonstrated the feasibility of mapping soft-tissue elasticity using ultrafast ultrasound tracking of shear-wave propagation [4]. However, the measured stiffness values can depend on the excitation method, tracking strategy, frequency content, assumed mechanical model, region of interest, and device-specific reconstruction algorithm [3,7,8]. Therefore, stiffness values obtained from different systems or measurement configurations are not always interchangeable, and this remains a critical concern when developing new elastography platforms or translating experimental methods into biological and clinical applications [7,9,10].

In addition to elasticity, biological tissues exhibit viscoelastic behavior, meaning that their apparent mechanical response depends on excitation frequency [11]. This frequency dependence is important because soft tissues are not purely elastic solids; they store and dissipate mechanical energy during wave propagation. Shear-wave dispersion methods have therefore been used to estimate both elasticity and viscosity-related parameters from the frequency-dependent behavior of shear waves [12,13]. The Kelvin–Voigt fractional derivative (KVFD) model and related rheological models are commonly used to describe the frequency-dependent viscoelastic response of soft tissues because they account for both elastic storage and viscous dissipation over a range of frequencies [12,14]. Such model-based analysis enables frequency-dependent shear-wave measurements to be represented using apparent viscoelastic parameters rather than a single stiffness value.

We previously developed a shear-wave time-of-flight (TOF) device and evaluated it in tissue-mimicking phantoms using frequency-dependent shear-wave-speed measurements and KVFD fitting [15]. In that phantom-based work, the TOF device estimated elasticity, viscosity, and fractional-order parameters under controlled material conditions supporting its potential as a calibration-oriented and research-focused platform [15]. However, phantom-based evaluation alone does not establish whether the device can be applied effectively to ex vivo biological tissues, where attenuation, coupling conditions, tissue handling, fluid loss, and structural organization may influence shear-wave generation, propagation, and detection [16,17].

Ex vivo biological tissues provide an important intermediate model between controlled phantom studies and more complex in vivo applications. They retain relevant mechanical and structural features of biological materials while allowing controlled access to the sample geometry, excitation direction, and measurement configuration [13]. Porcine liver and chicken breast muscle were selected because they represent distinct tissue classes: liver as a soft parenchymal tissue and chicken breast as a skeletal muscle with an organized fiber structure [18]. In skeletal muscle, shear-wave measurements depend on the direction of propagation relative to muscle fibers, and previous elastography studies have shown that fiber alignment influences the measured mechanical response [19,20]. Measuring chicken breast parallel and perpendicular to the visible muscle fibers therefore provides a practical means of assessing whether a TOF device can detect direction-dependent shear-wave responses.

The present study extends the previously evaluated phantom-based TOF approach to ex vivo porcine liver and chicken breast muscle. For biological tissue measurements, the transmitting architecture was modified to use a woofer-based electrodynamic actuator for low-frequency mechanical excitation, while a piezoelectric bimorph was retained as the receiving sensor because of its sensitivity to small dynamic tissue vibrations.

The main objective of this feasibility study was to determine whether the shear-wave TOF platform previously evaluated by our group in tissue-mimicking phantoms could be applied to ex vivo biological tissues. Specifically, the study assessed TOF detectability, frequency-dependent apparent shear-wave propagation speed, KVFD model-dependent fit parameters, signal quality, and direction-dependent responses in chicken breast measured parallel and perpendicular to the visible muscle fibers.

A preliminary descriptive comparison with the HISKY EQTouch UD3000 SWE system was also performed as a practical stiffness-range check rather than a validation analysis, because the two systems differ in excitation mechanism, frequency content, sensing and signal-processing approaches, and underlying mechanical assumptions [9,15]. Overall, the study provides a focused feasibility assessment of TOF-based shear-wave measurement in ex vivo biological tissues.

## 2. Materials and Methods

### 2.1. Study Design

This study was designed as an ex vivo feasibility and tissue-sensitivity assessment of a custom shear-wave TOF device for biological tissue measurement. The experimental design included three measurement conditions: porcine liver, chicken breast measured parallel to the muscle-fiber direction, and chicken breast measured perpendicular to the muscle-fiber direction. Three porcine liver samples and three chicken breast samples were evaluated. The same three chicken breast samples were measured in both the parallel and perpendicular orientations, resulting in paired orientation measurements. Tissue samples were treated as the sample-level observations, whereas excitation frequencies and repeated excitation shots were treated as measurement conditions and technical repetitions, respectively.

The primary measured outcomes were TOF-derived apparent shear-wave speed, frequency-dependent propagation behavior, signal-quality metrics, and direction-dependent TOF responses. Frequency-dependent apparent shear-wave speeds were subsequently fitted using the KVFD model to obtain model-dependent fit parameters, including the elastic fi parameter *E*_0_. TOF measurements across all conditions were performed using the same signal-processing workflow, frequency range, and onset-detection algorithm. Direction-dependent analysis was performed only for chicken breast muscle, because skeletal muscle has a visible fiber orientation and can be measured with the propagation path aligned either parallel or perpendicular to the fibers [21,22,23].

A preliminary descriptive comparison was also performed between the KVFD elastic fit parameter *E*_0_ and stiffness values reported by the HISKY EQTouch UD3000 healthcare elastography device. This comparison was restricted to porcine liver and chicken breast measured parallel to the muscle fibers. It was used only as a practical stiffness-range check and was not treated as a validation or agreement analysis.

### 2.2. Tissue Samples and Preparation

Fresh ex vivo porcine liver and chicken breast tissues were used. Porcine liver was selected as a soft parenchymal tissue, whereas chicken breast was selected as a skeletal muscle tissue with a visible fiber orientation. Three samples were evaluated for each tissue type. Each of the three chicken breast samples was measured in both propagation orientations, resulting in paired measurements from the same samples.

The tissues were obtained on the day of measurement and tested approximately 5 h after procurement. Before measurement, the tissue samples were kept in phosphate-buffered saline (PBS) to reduce dehydration during handling and preparation [24]. Immediately before testing, each sample was removed from PBS, gently blotted to remove excess surface fluid, and positioned in the TOF measurement platform. All measurements were performed at room temperature (approximately 23 °C).

Porcine liver samples were measured without directional alignment because no dominant fiber direction was assigned to this tissue. For each chicken breast sample, the visible muscle-fiber direction was identified before measurement. Each sample was measured with the shear-wave propagation path oriented both parallel and perpendicular to the visible muscle fibers, with a 90° reorientation between measurements. After each chicken breast sample was reoriented, the Tx–Rx propagation distance was remeasured using a caliper and maintained at 23.52 mm before data acquisition. The same tissue-preparation, hydration, and elapsed-measurement-time protocol was applied to both orientations. To reduce systematic order effects, the starting orientation was alternated across samples: samples measured first in the parallel orientation were followed by samples measured first in the perpendicular orientation. The orientation protocol therefore comprised two paired chicken breast measurement conditions: propagation parallel and perpendicular to the muscle-fiber direction. Table 1 summarizes the tissue samples and preparation and measurement conditions.

### 2.3. TOF Device and Experimental Setup

The shear-wave TOF measurement system consisted of a mechanical excitation pathway, a biological tissue sample, a receiving piezoelectric sensor, a Rigol MSO8064 digital oscilloscope (RIGOL Technologies Co., Ltd., Suzhou, China), and MATLAB 2024b (MathWorks, Inc., Natick, MA, USA)-based post-processing. The excitation pathway included a DDS/function generator, a TPA3116D2 (Texas Instruments Inc., Dallas, TX, USA) Class-D power amplifier, a SW40LLB-01 electrodynamic woofer, and a lightweight 3D-printed connector attached to the woofer dust cap. The DDS/function generator generated the excitation signal, which was amplified by the TPA3116D2 power amplifier and delivered to the 4-inch SW40LLB-01 woofer, which converted the electrical excitation into low-frequency mechanical vibration. The 3D-printed connector weighed approximately 1.3 g and was used to transfer mechanical excitation from the woofer to the tissue sample.

The receiving side consisted of a piezoelectric bimorph sensor positioned on the opposite side of the tissue sample along the intended shear-wave propagation path. The excitation connector and receiving bimorph were aligned so that the generated mechanical disturbance propagated through the tissue from the excitation side to the receiving side. The Tx–Rx propagation distance was 22.42 mm for porcine liver and 23.52 mm for chicken breast. For chicken breast, the same Tx–Rx distance was used for both the parallel and perpendicular muscle-fiber orientations.

The excitation reference signal was recorded using channel 1 of the Rigol MSO8064 oscilloscope, whereas the received tissue response from the piezoelectric bimorph was recorded using channel 2. This two-channel acquisition allowed the excitation reference and received response to be recorded synchronously for TOF estimation. The acquired signals were exported to MATLAB for signal preprocessing, onset detection, shear-wave-speed calculation, frequency-dependent analysis, and model fitting.

We previously evaluated the underlying TOF methodology in tissue-mimicking phantoms through controlled multi-distance measurements, assessment of propagation-distance dependence, repeatability testing, and comparison with rheometry [15]. In the present study, the transmitting architecture was modified to use a woofer-based electrodynamic actuator to provide stronger low-frequency mechanical excitation in biological tissues. Because the frequency-dependent electromechanical delay of this modified excitation pathway was not independently calibrated, a residual system-delay contribution could not be excluded. Accordingly, the propagation speeds were interpreted as apparent quantities, and the KVFD outputs were treated as model-dependent fit parameters.

**Figure 1:**
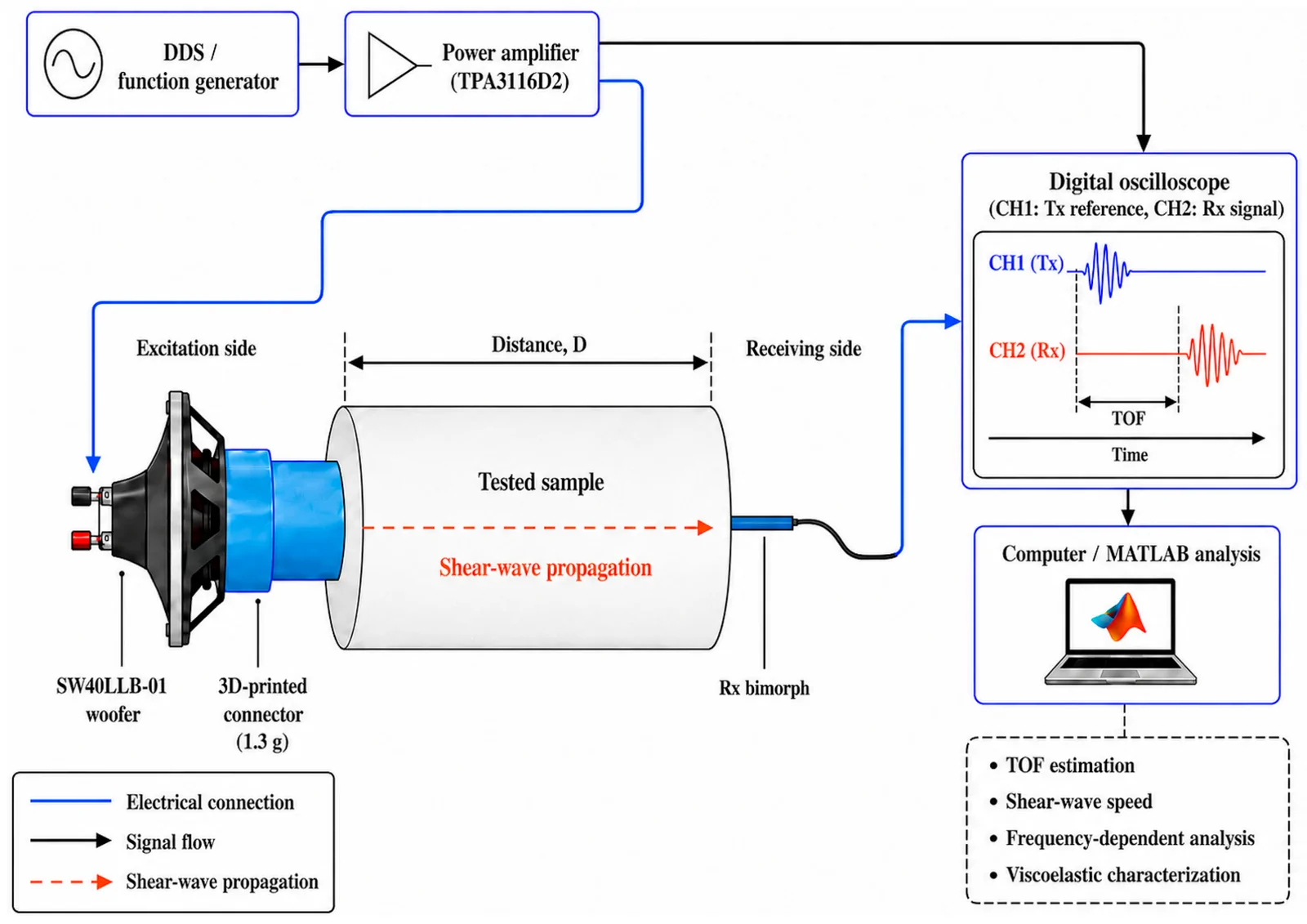
Schematic of the shear-wave TOF device and experimental setup.

The overall device configuration and signal-flow pathway are shown in Figure 1. The setup was designed to provide controlled low-frequency mechanical excitation while allowing synchronous measurement of the temporal delay between the excitation reference and the received tissue response.

### 2.4. Excitation Protocol and Data Acquisition

Shear-wave measurements were performed using one-cycle sinusoidal excitation bursts. Seven excitation frequencies were tested for each tissue condition: 40, 60, 80, 100, 120, 140, and 160 Hz. The same frequency range was used for porcine liver, chicken breast measured parallel to the muscle fibers, and chicken breast measured perpendicular to the muscle fibers. The seven excitation frequencies represented measurement conditions and were not treated as statistical replicates.

For each tissue sample, applicable measurement orientation, and excitation frequency, 50 excitation shots and their corresponding received responses were acquired. The 50 Tx and Rx waveforms were averaged before signal processing, producing one averaged Tx waveform and one averaged Rx waveform for each sample–orientation–frequency combination. The averaged Tx waveform was used as the transmitted reference signal, whereas the averaged Rx waveform was used for Rx onset detection, TOF estimation, shear-wave-speed calculation, and frequency-dependent analysis. The 50 excitation shots were treated as technical repetitions used to improve waveform stability and were not regarded as independent statistical observations.

Synchronized two-channel acquisition was performed using a Rigol MSO8064 digital oscilloscope. Channel 1 recorded the transmitted reference signal from the excitation pathway, and channel 2 recorded the received response from the piezoelectric bimorph sensor. During acquisition, the oscilloscope operated at a sampling rate of 20 kSa/s with a memory depth of 10 kpts and a horizontal time scale of 50 ms/div. These acquisition settings allowed both the transmitted excitation and the delayed received tissue response to be captured within the same recording window. For each frequency and tissue condition, the acquired data were exported to MATLAB as an averaged time vector, averaged Tx signal, and averaged Rx signal. These variables were stored as tt, txAvg, and rxAvg, respectively, and used as input data for the signal-processing pipeline. The same excitation protocol, acquisition settings, and post-processing workflow were applied across all TOF measurement conditions to ensure consistency between porcine liver, chicken breast parallel, and chicken breast perpendicular measurements.

### 2.5. Signal Preprocessing and TOF Detection

Signal preprocessing and TOF detection were performed in MATLAB using the averaged Tx and Rx waveforms exported from the oscilloscope. The same processing pipeline was applied to all tissue conditions and excitation frequencies. For each measurement, the input data comprised the time vector, the averaged transmitted reference signal, and the averaged received signal. The effective sampling frequency was calculated from the median interval of the exported time vector and was approximately 20 kSa/s.

Before onset detection, the Tx and Rx signals were preprocessed to reduce impulsive noise, out-of-band components, and baseline drift. A median filter with a five-sample window was first applied to both signals. Following median filtering, the signals were processed using a frequency-adaptive finite-impulse-response (FIR) filter. The filter bandwidth was defined as $BW=\max\left(20,2f_{0}/N_{\mathrm{cycles}}\right)\;\mathrm{Hz}$, where *f*_0_ is the excitation frequency and N_cycles_ = 1 is the number of transmitted cycles. The nominal lower and upper cut-off frequencies were calculated as $f_{L}=\max\left(1,f_{0}-BW/2\right)$ and $f_{H}=\min\left(f_{s}/2-1,f_{0}+BW/2\right)$ respectively. A 50th-order, 51-coefficient Hamming-window FIR filter was designed in MATLAB using fir1 and applied to both signals by forward–backward filtering using filtfilt.

Because a single-cycle burst was used at every excitation frequency, the 20 Hz minimum bandwidth was not activated, and the nominal filter ranges extended from approximately 1–80 Hz at 40 Hz excitation to 1–320 Hz at 160 Hz excitation. Owing to the low normalized cut-off frequencies relative to the sampling frequency and the limited filter order, the realized responses were broad and low-pass-like rather than sharply selective bandpass responses. The filter was therefore used to suppress higher-frequency noise while retaining the broadband spectral content of the one-cycle excitation, rather than to isolate a fixed 20 Hz frequency band. The calculated single-pass and effective forward–backward magnitude responses for all tested excitation frequencies are provided in Supplementary Figure S1.

Following FIR filtering, the signals were smoothed using a third-order Savitzky–Golay filter with a 51-sample frame length, and the Rx signal was linearly detrended. The Tx onset was detected directly from the transmitted reference signal, whereas the fully processed and detrended Rx signal was used for cumulative-energy onset detection.

The baseline level was estimated from the initial 20 ms noise segment and subtracted from the Tx waveform. The absolute value of the baseline-corrected Tx signal was then calculated. A robust Tx threshold was defined as the larger of a median absolute deviation-based threshold and 10% of the maximum Tx amplitude:

(1) $$T_{Tx}=\max\left[m_{0}+8\left(1.4826,MAD_{0}\right),0.10\max\left(\left|x_{Tx}\right|\right),\right.$$

where $\left(m_{0}\right)$ is the median of the absolute Tx signal within the baseline segment, $\left(MAD_{0}\right)$ denotes its median absolute deviation and $\left(x_{Tx}\right)$ is the baseline-corrected Tx waveform. The factor 1.4826 provides a standard-deviation (SD)-equivalent estimate under normally distributed noise, consistent with the normal-consistency scaling used for MAD-based robust scale estimation [25,26].

The first sustained threshold crossing was selected as the Tx onset, $\left(t_{Tx}\right)$. Short threshold-crossing events were rejected using the minimum run-length criterion:

(2) $$N_{run}=\max\left[5,round\left(0.08\frac{f_{s}}{f}\right)\right]$$

The Rx onset was detected using a cumulative-energy approach applied to the processed Rx signal. For the received signal $\left(x\left(t\right)\right)$, the analytic signal was calculated using the Hilbert transform:

(3) $$z\left(t\right)=x\left(t\right)+jH\{x\left(t\right)\}$$

where $\left(Hx\left(t\right)\right)$ denotes the Hilbert transform of $\left(x\left(t\right)\right)$. The signal envelope was then calculated as:

(4) $$A\left(t\right)=\left|z\left(t\right)\right|$$

and the envelope-energy signal was defined as:

(5) $$E\left(t\right)=A^{2}\left(t\right)$$

The envelope-energy signal was smoothed using a frequency-dependent moving-average window before cumulative integration. The cumulative energy was calculated as:

(6) $$C\left(t\right)=\int_{t_{0}}^{t}E\left(\tau \right),d\tau$$

where $\left(t_{0}\right)$ is the beginning of the analyzed signal segment. To reduce the influence of baseline drift, a linear trend fitted to the cumulative energy within the initial 20 ms noise segment was subtracted:

(7) $$C_{c}\left(t\right)=C\left(t\right)-C_{baseline}\left(t\right)$$

The derivative of the corrected cumulative-energy curve was then calculated:

(8) $$S\left(t\right)=\frac{dC_{c}\left(t\right)}{dt}$$

The derivative signal was additionally smoothed using a 0.2 ms moving-average window to stabilize the slope criterion. The Rx search started 0.5 ms after the detected Tx onset. The envelope-energy smoothing window was defined as $w_{E}=\max\left[1.0,\min\left(2.0,0.12T\right)\right]$ ms, and the minimum sustained-crossing duration was defined as $w_{s}=\max\left[1.5,\min\left(3.0,0.20T\right)\right]$ ms, where T = 1000/f0 is the excitation period in milliseconds.

The cumulative-energy threshold was selected as the larger of the baseline-derived threshold and a relative threshold equal to 1% of the maximum corrected cumulative energy within the post search region.

(9) $$T_{C}=\max\left[\mu_{C}+10\sigma_{C},0.01\;\max_{t\text{∈}\Omega_{s}}C_{c}\left(t\right)\right]$$

where $\left(\mu_{C}\right)$ and $\left(\sigma_{C}\right)$ are the mean and SD of $C_{c}\left(t\right)$ within the baseline segment, and $\Omega_{s}$ denotes the Rx search region. The derivative threshold was calculated as:

(10) $$T_{S}=\mu_{S}+10\sigma_{S}$$

where $\left(\mu_{S}\right)$ and $\left(\sigma_{S}\right)$ are the mean and SD of $S\left(t\right)$ within the baseline segment.

The Rx onset was defined by the simultaneous conditions:

(11) $$C_{c}\left(t\right)>T_{C},\;and\;S\left(t\right)>T_{S}$$

The Rx onset, $\left(t_{Rx}\right)$, was assigned to the first acquired sample of the earliest threshold crossing for which both conditions remained satisfied throughout the required sustained duration. A valid detection required $t_{\mathrm{Rx}}>t_{\mathrm{Tx}}$. If no crossing satisfied both criteria for the required duration, the first crossing of the corrected cumulative-energy threshold within the Rx search region was used as a fallback onset estimate. The TOF at each excitation frequency was calculated as:

(12) $$TOF\left(f\right)=t_{Rx}\left(f\right)-t_{Tx}\left(f\right)$$

where $\left(t_{Tx}\left(f\right)\right)$ and $\left(t_{Rx}\left(f\right)\right)$ are the detected Tx and Rx onset times at excitation frequency $\left(f\right)$, respectively. The resulting TOF values were used to calculate shear-wave speed and to perform frequency-dependent analysis.

The Rx onset-detection procedure was validated using one-cycle synthetic bursts at the seven tested excitation frequencies with imposed delays of 2.5, 4.0, 6.0, 9.2, 14.0, and 20.0 ms. White Gaussian noise was added at SNRs of 10, 20, 30, and 40 dB. Twenty independent noise realizations were analyzed for each frequency–delay–SNR combination, yielding 3360 validation trials. Each synthetic waveform was processed using the same Rx median filtering, forward–backward FIR filtering, Savitzky–Golay smoothing, detrending, and cumulative-energy onset-detection procedure applied to the experimental signals. The validation results are presented in the Supplementary Table S1.

The sampling interval at approximately 20 kSa/s was approximately 0.05 ms. The Tx and Rx onset times were assigned directly to the first acquired samples satisfying their respective sustained detection criteria. No sub-sample interpolation, resampling, or curve-fitting procedure was applied to refine the onset times. Consequently, the temporal resolution of each individual TOF estimate was limited to approximately one sampling interval.

### 2.6. TOF-Derived Apparent Shear-Wave Propagation Speed

The TOF-derived apparent shear-wave propagation speed was calculated from the known Tx–Rx propagation distance and the detected onset-time difference:

(13) $$c_{app}\left(f\right)=\frac{D}{TOF\left(f\right)}$$

where $\left(c_{app}\left(f\right)\right)$ is the apparent shear-wave speed (SWS) at excitation frequency $\left(f\right),\left(D\right)$ is the Tx–Rx propagation distance, and $\left(TOF\left(f\right)\right)$ is the measured time of flight at that frequency.

Because the Rx onset was determined from the first sustained increase in the cumulative energy of the received waveform, $\left(c_{app}\left(f\right)\right)$ represents an onset-based propagation-speed estimate rather than a direct measurement of phase velocity. In addition, residual electromechanical system delay and the systematic onset-timing bias identified during validation could not be completely separated from the tissue propagation time. The calculated values are therefore reported as apparent shear-wave speeds.

A Tx–Rx distance of 22.42 mm was used for porcine liver, whereas a distance of 23.52 mm was used for chicken breast. For each chicken breast sample, the same nominal distance was maintained for the parallel and perpendicular muscle-fiber orientations and was remeasured after sample reorientation.

Apparent shear-wave speeds were calculated at excitation frequencies of 40, 60, 80, 100, 120, 140, and 160 Hz. The resulting frequency-dependent apparent propagation-speed data were used to assess tissue- and direction-dependent behavior and as inputs to the KVFD fitting procedure. Because these data were derived from onset-based TOF measurements rather than direct phase-velocity measurements, the resulting KVFD parameters were interpreted as model-derived apparent viscoelastic estimates rather than absolute material properties.

For each tissue condition and excitation frequency, the apparent shear-wave speed was reported as mean ± SE across the three biological samples; therefore, the SE represents between-sample variability. Each sample-level value was calculated from a TOF estimate determined at the native temporal resolution of approximately 0.05 ms. A first-order resolution-based uncertainty estimate was obtained from $c_{\mathrm{app}}=D/\mathrm{TOF}$ as $u_{c_{\mathrm{app}}}/c_{\mathrm{app}}=\sqrt{{\left(u_{D}/D\right)}^{2}+{\left(u_{\mathrm{TOF}}/\mathrm{TOF}\right)}^{2}}$, using the caliper resolution of 0.01 mm and the temporal sampling interval of 0.05 ms. The resulting relative uncertainty was approximately 0.6–0.9% for porcine liver and 1.0–1.5% for chicken breast. Because sample-level speeds were subsequently averaged, the resulting group means were not restricted to increments corresponding directly to a single sampling interval. Systematic onset-timing bias, residual system delay, and coupling effects were considered separately in Section 2.5 and Section 4.2.

### 2.7. KVFD Fitting and Model-Dependent Parameter Estimation

Frequency-dependent TOF-derived apparent shear-wave-speed data were fitted using the Kelvin–Voigt fractional derivative (KVFD) model [24,27]. The KVFD model provides a physically motivated constitutive description of viscoelastic behavior, in which elastic storage and viscous dissipation jointly determine the frequency-dependent complex modulus. Combined with the shear-wave propagation relation, it provides an analytical link between tissue viscoelasticity and the observed frequency-dependent apparent propagation speed. Because the fitted quantity was an onset-based apparent propagation speed rather than a directly measured phase velocity, the KVFD model was used here as a descriptive framework for the observed frequency dependence rather than as a complete representation of the transient wave field, and the fitted parameters were therefore treated strictly as model-dependent fit parameters and not as direct estimates of constitutive material properties.

In the KVFD formulation, the complex Young’s modulus is expressed as:

(14) $$E^{*}\left(\omega \right)=E_{0}+\eta {\left(j\omega \right)}^{\alpha }$$

where $\left(E_{0}\right)$ is the elastic parameter, $\left(\eta \right)$ is the viscosity-related parameter, $\left(\alpha \right)$ is the fractional derivative order, $\left(j=\sqrt{-1}\right)$, and $\left(\omega =2\pi f\right)$ is the angular frequency. Assuming soft tissue to be nearly incompressible, the complex shear modulus was related to the complex Young’s modulus by:

(15) $$G^{*}\left(\omega \right)=\frac{E^{*}\left(\omega \right)}{3}$$

The corresponding KVFD-predicted apparent shear-wave speed was calculated as:

(16) $$c_{s}\left(f\right)=\sqrt{\frac{2Q\left(f\right)}{3\rho \left[\sqrt{Q\left(f\right)}+E_{0}+\eta \cos\left(\frac{\pi \alpha }{2}\right){\left(2\pi f\right)}^{\alpha }\right]}}$$

where

$$Q\left(f\right)=E_{0}^{2}+2E_{0}\eta \cos\left(\frac{\pi \alpha }{2}\right){\left(2\pi f\right)}^{\alpha }+\eta^{2}{\left(2\pi f\right)}^{2\alpha }$$

and $\left(\rho \right)$ is the assumed tissue density, which was set to $\left(1000\;kg/m^{3}\right)$ for all samples.

The KVFD model was fitted separately to each tissue sample using its seven frequency-specific apparent shear-wave-speed values from 40 to 160 Hz. Parameter estimation was performed by minimizing:

(17) $$SSE=\sum_{i=1}^{n}{\left[c_{s,meas}\left(f_{i}\right)-c_{s,model}\left(f_{i};E_{0},\eta ,\alpha \right)\right]}^{2}$$

where $\left(c_{s,meas}\left(f_{i}\right)\right)$ and $\left(c_{s,model}\left(f_{i};E_{0},\eta ,\alpha \right)\right)$ are the measured and model-predicted apparent shear-wave speeds, respectively, and n = 7 is the number of tested frequencies.

The *E*_0_, *η*, and *α* were estimated by bounded, unweighted nonlinear least squares using MATLAB lsqnonlin. The nominal initial values were *E*_0_ = 3 kPa, *η* = 10 kPa·s^α^, and *α* = 0.3, with bounds of 3 kPa–10 Mpa, 100 Pa·s^α^–1 M Pa·s^α^, and 0.05–0.95, respectively. One nominal and 300 random starting sets were evaluated; *E*_0_ and *η* were sampled logarithmically and *α* uniformly within their respective bounds. The maximum numbers of iterations and function evaluations were both 104, and the converged solution with the lowest residual sum of squares was retained.

For each sample-level fit, R^2^ and RMSE were calculated from the same seven measured and model-predicted apparent shear-wave-speed values. Three sample-level R^2^ values and three sample-level RMSE values were therefore obtained for each tissue-orientation condition. These values were then averaged to provide the condition-level goodness-of-fit metrics reported in Table 2; the reported R^2^ and RMSE values were not calculated from fits to group-averaged shear-wave-speed curves.

Sample-level parameters were summarized as mean ± SE across the three samples for each tissue condition. The fitted *E*_0_, *η*, and *α* were reported as model-dependent KVFD fit parameters. Because the three parameters were jointly estimated from seven frequencies over a restricted bandwidth, no claim of unique parameter identification was made.

### 2.8. Directional-Dependent TOF Response

Direction-dependent shear-wave responses were evaluated in chicken breast muscle by comparing paired measurements obtained with the propagation path oriented parallel and perpendicular to the visible muscle-fiber direction. The same three chicken breast samples were evaluated in both orientations. After each sample was reoriented, the Tx–Rx propagation distance was remeasured using a caliper and maintained at 23.52 mm. The excitation frequencies, acquisition settings, and signal-processing pipeline were kept unchanged between orientations, and the starting orientation was alternated across samples to reduce systematic measurement-order effects. However, contact force and tissue compression at the actuator and receiver interfaces were not quantitatively measured or controlled.

For the parallel orientation, the tissue was positioned so that the shear-wave propagation path followed the visible muscle-fiber direction. For the perpendicular orientation, the same sample was reoriented by 90° so that the propagation path crossed the muscle fibers. This design was used because skeletal muscles are structurally organized and shear-wave propagation can depend on fiber alignment.

At each excitation frequency, the shear-wave speed measured parallel to the muscle fibers was compared with the shear-wave speed measured perpendicular to the muscle fibers. The percentage difference between orientations was calculated as:

(18) $$\mathrm{Difference}=\left(\frac{c_{∥}\left(f\right)-c_{⊥}\left(f\right)}{c_{∥}\left(f\right)}\right)\times 100\%$$

where $c_{∥}\left(f\right)$ is the shear-wave speed measured parallel to the muscle fibers and $c_{⊥}\left(f\right)$ is the shear-wave speed measured perpendicularly to the muscle fibers at excitation frequency $\left(f\right)$. A positive value indicated that the perpendicular speed was lower than the parallel speed.

Parallel-to-perpendicular apparent speed ratio was calculated using the mean shear-wave speed across the tested frequency range:

(19) $$AR_{c}=\frac{\bar{c_{∥}}}{\bar{c_{⊥}}}$$

where $c_{∥}\left(f\right)$ and $c_{⊥}\left(f\right)$ are the mean shear-wave speeds from 40 to 160 Hz for the parallel and perpendicular orientations, respectively.

Direction-dependent variation in the KVFD elastic fit parameter, *E*_0_, was assessed by comparing the values obtained from the paired parallel and perpendicular measurements. The parallel-to-perpendicular *E*_0_ ratio was calculated as

(20) $$R_{E_{0}}=\frac{E_{0,⊥}}{E_{0,∥}}$$

where $E_{0,∥}$ and $E_{0,⊥}$ are the KVFD elastic fit parameter values obtained from chicken breast measurements with propagation parallel and perpendicular to the muscle fibers, respectively. Because *E*_0_ was obtained by fitting the KVFD relation to onset-based apparent propagation-speed data, it was treated strictly as a model-dependent fit parameter rather than as a direct estimate of Young’s modulus or tissue stiffness. Furthermore, because contact force and local tissue compression were not quantitatively controlled and independent mechanical anisotropy testing was not performed, the observed parallel-to-perpendicular differences were interpreted as direction-dependent TOF responses under the tested experimental configuration rather than as definitive evidence of intrinsic muscle anisotropy.

### 2.9. Signal-Quality Analysis

Signal quality and detection performance were assessed for porcine liver and chicken breast measured with propagation parallel and perpendicular to the muscle fibers. The analysis used the processed Rx waveform after filtering, smoothing, and detrending. The evaluated metrics included peak-envelope amplitude, signal-to-noise ratio (SNR), and TOF detection performance.

The received peak-envelope amplitude was calculated from the Hilbert envelope of the processed Rx waveform. Following detection of a valid Rx onset, a post-onset signal window with a duration equal to one excitation period was selected. The maximum envelope amplitude within this window was defined as:

(21) $$A_{\mathrm{peak}}=\max\{e_{\mathrm{Rx}}\left(t\right):t\in \Omega_{s}\}$$

where $e_{\mathrm{Rx}}\left(t\right)$ is the Hilbert-envelope amplitude of the processed Rx waveform and $\Omega_{s}$ is the post-onset signal window. The signal root-mean-square amplitude, $\mathrm{RM}S_{\mathrm{signal}}$, was calculated within the same window, whereas the noise root-mean-square amplitude, $\mathrm{RM}S_{\mathrm{noise}}$, was calculated from the pre-excitation baseline segment preceding the Tx onset. SNR was calculated as:

(22) $$SNR_{dB}=20\;\log_{10}\left(\frac{RMS_{signal}}{RMS_{noise}}\right)$$

The reported SNR was calculated from filtered, 50-shot ensemble-averaged Rx waveforms and therefore reflects post-processing SNR rather than single-shot SNR. Under ideal coherent averaging with independent incoherent noise, 50-shot averaging provides a theoretical SNR gain of approximately 17 dB. Because the individual excitation responses were not retained, single-shot SNR and shot-to-shot TOF variability could not be determined retrospectively.

Detection performance was assessed across all sample–frequency measurements. A primary detection was classified as successful when valid Tx and Rx onsets were identified, the Rx onset occurred after the Tx onset, the sustained cumulative-energy and slope-threshold criteria were satisfied without use of the fallback criterion, and the derived apparent shear-wave speed was within the predefined analysis range of 1–10 m/s. This range corresponded to TOFs of approximately 2.2–23.5 ms over the Tx–Rx distances used. The primary-detection rate was calculated as

(23) $$Detection\;success\;rate=\frac{N_{primary}}{N_{total}}\times 100\%$$

where $N_{\mathrm{primary}}$ is the number of measurements satisfying all primary-detection criteria and $N_{\mathrm{total}}$ is the total number of sample–frequency measurements. Each tissue condition comprised 21 measurements, three samples evaluated at seven frequencies.

Signal-quality metrics were calculated at excitation frequencies of 40–160 Hz. For each tissue condition, received peak-envelope amplitude and SNR were summarized as mean ± SD across the seven frequency-specific sample means. The 10 dB SNR reference line was included only for visual interpretation of the signal-quality plots and was not used as an exclusion criterion.

### 2.10. UD3000 Preliminary Comparison Protocol

A preliminary descriptive comparison was performed between the KVFD elastic fit parameter *E*_0_ and stiffness values reported by Hisky EQTouch UD3000. UD3000 measurements were performed on porcine liver and chicken breast muscle using a 7.5M/L50/192 linear-array probe with a frequency range of 5–12 MHz and a nominal center frequency of 7.5 MHz.

To reduce operator-dependent variability, the probe was mounted vertically in a mechanical clamp, and the ultrasound beam was aligned perpendicular to the tissue surface. After confirming stable B-mode visualization, the system was switched to SWE mode. A rectangular region of interest (ROI) of 10 × 15 mm was positioned at the central region of the tissue. The measurement depth was adjusted to account for tissue thickness while maintaining clearance from the tissue boundaries.

Repeated SWE measurements were acquired at different locations within the ROI. The measurement diameter was 1.3 mm. UD3000 stiffness values were recorded as Young’s modulus in kilopascals. The repeated UD3000 values were summarized as mean ± SD for each tissue condition.

The corresponding TOF-derived apparent Young’s modulus was obtained by fitting the frequency-dependent apparent shear-wave speeds using the KVFD model. The absolute difference between TOF-derived apparent Young’s modulus and the UD3000 SWE stiffness measurement was calculated as:

(24) $$\Delta E=\left|E_{TOF}-E_{UD3000}\right|$$

where $\left(E_{TOF}\right)$ is the TOF-derived apparent Young’s modulus and $\left(E_{UD3000}\right)$ is the mean Young’s modulus measured by UD3000 SWE.

Because neither method was treated as an absolute reference standard, the relative difference was calculated using the symmetric percentage difference:

(25) $$SPD=\frac{\left|E_{TOF}-E_{UD3000}\right|}{\frac{\left(E_{TOF}+E_{UD3000}\right)}{2}}\times 100\%$$

This comparison was interpreted descriptively as a preliminary stiffness-range comparison, not as validation or agreement analysis.

## 3. Results

### 3.1. TOF Detection and Received Shear-Wave Identification in Ex Vivo Tissue

**Figure 2:**
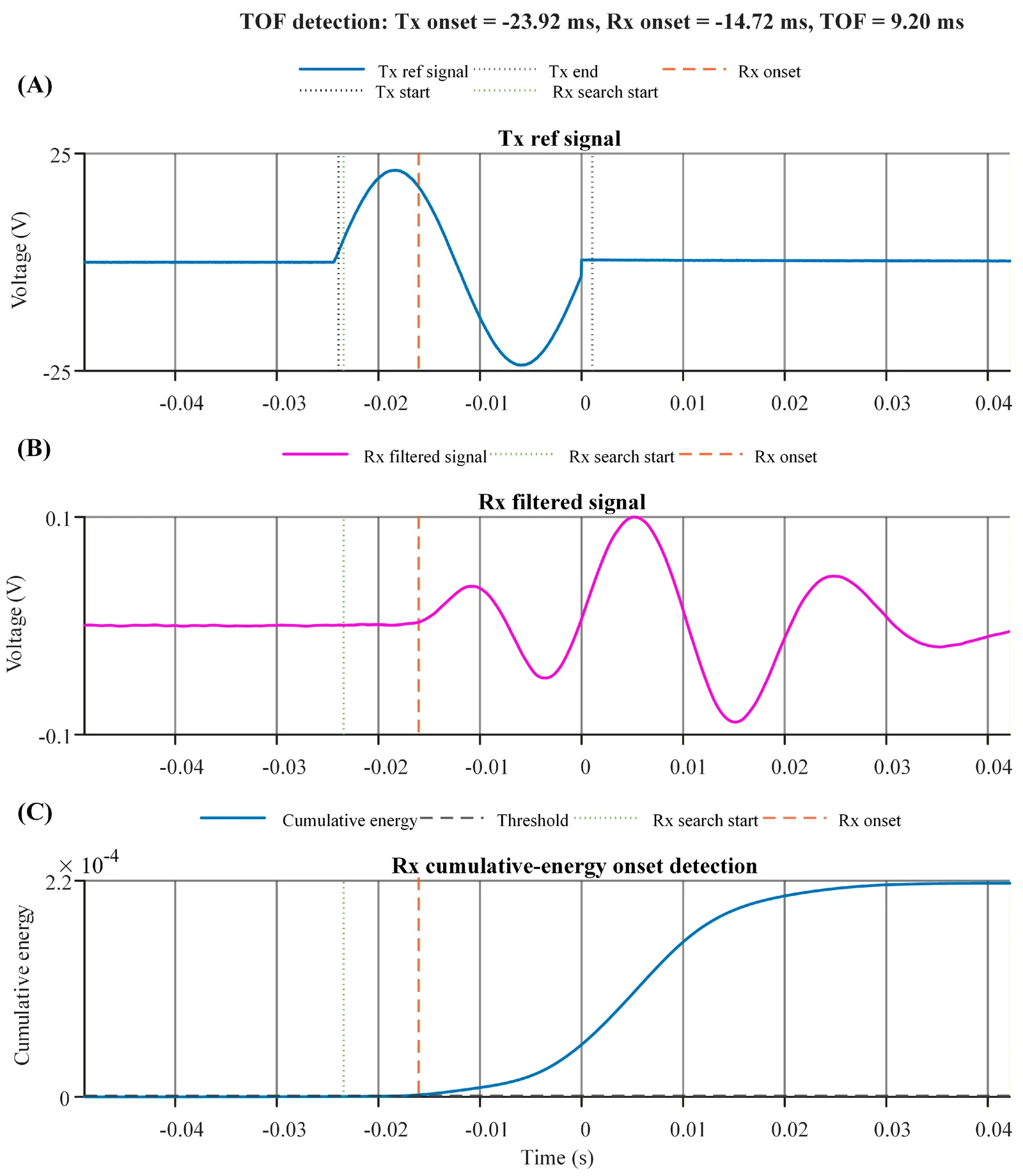
Representative TOF detection workflow for an ex vivo porcine liver measurement at 40 Hz. (**A**) Transmitted reference signal showing the detected Tx onset and Tx end. (**B**) Filtered received signal showing the Rx search start and detected Rx onset. (**C**) Baseline-corrected cumulative-energy curve used for Rx onset detection. The vertical dashed lines indicate the onset points used for TOF calculation.

Representative TOF detection from an ex vivo porcine liver measurement at 40 Hz is shown in Figure 2. The transmitted reference signal provided a clear excitation onset, while the filtered received signal showed a delayed response after propagation through the tissue. The cumulative-energy curve increased after the transmitted excitation, allowing the Rx onset to be identified from the first sustained rise above the noise-derived threshold.

As shown in Figure 2, the algorithm detected the Tx onset at −23.92 ms and the Rx onset at −14.72 ms, yielding a TOF of 9.20 ms. Using the Tx–Rx propagation distance of 22.42 mm, the corresponding shear-wave speed was 2.438 m/s, consistent with the mean 40 Hz porcine liver speed reported in Table A1. The representative result demonstrates that the cumulative-energy method can identify the received shear-wave arrival from processed ex vivo tissue signals without manual peak selection.

The same TOF detection pipeline was applied to porcine liver, chicken breast parallel to the muscle fibers, and chicken breast perpendicular to the muscle fibers across the tested frequency range of 40–160 Hz.

### 3.2. Onset-Detection Validation

**Figure 3:**
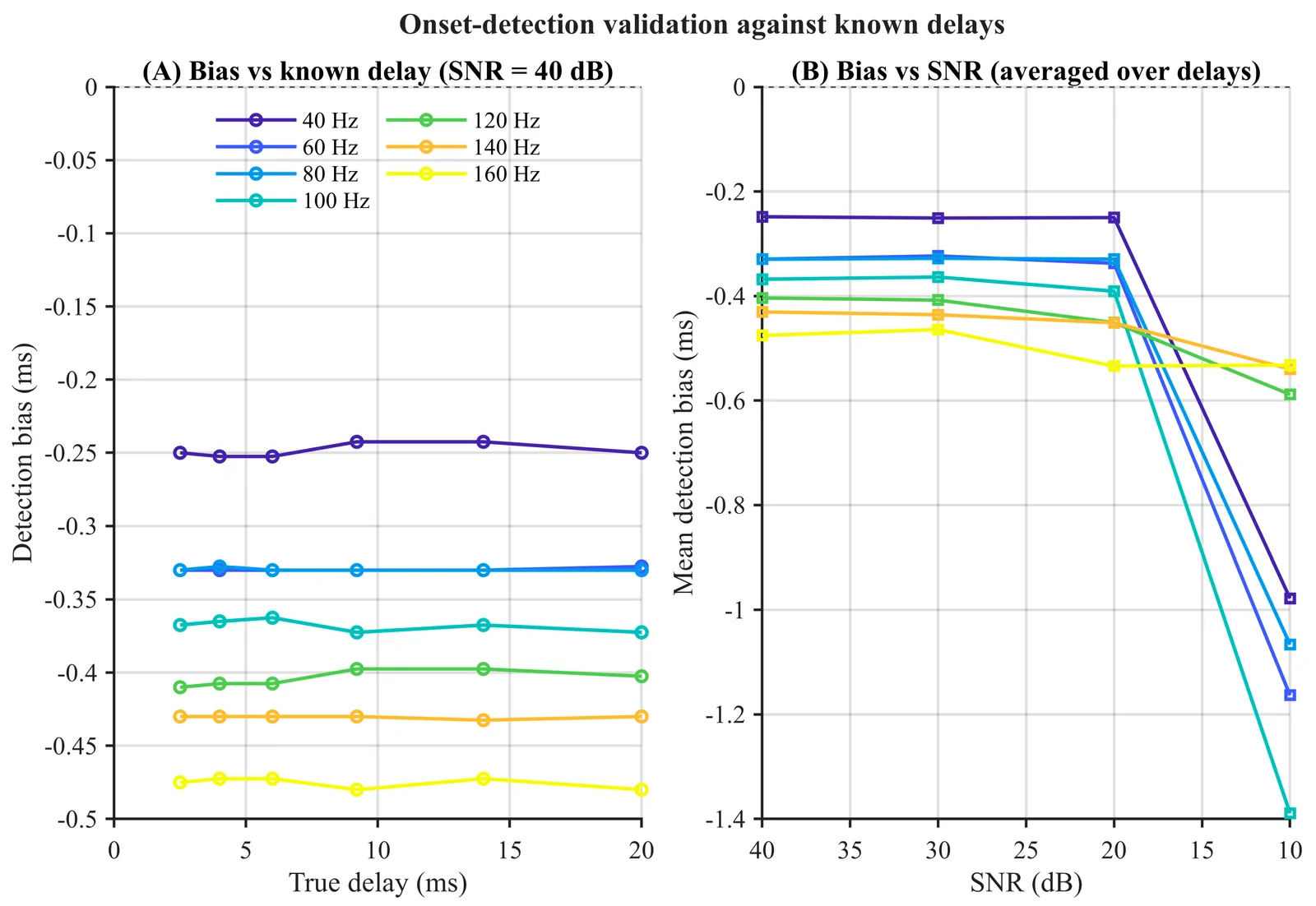
Validation of the Rx cumulative-energy onset-detection procedure using signals with known arrival times. (**A**) Mean onset bias as a function of imposed delay at 40 dB SNR for the seven tested excitation frequencies; each point represents 20 noise realizations. (**B**) Mean onset bias as a function of SNR, averaged across the six imposed delays; each point summarizes 120 trials. In both panels, line color denotes excitation frequency (40–160 Hz) as indicated by the legend in (**A**). Negative values indicate detection earlier than the imposed arrival time.

Validation using synthetic signals with known arrival times showed a systematic early-onset bias. At SNRs of 20–40 dB, the condition-specific mean bias ranged from −0.745 to −1.370 ms, where negative values indicate detection earlier than the imposed arrival time. At fixed frequency and SNR, the bias was largely independent of the imposed delay. At 40 Hz and 40 dB, for example, the mean bias varied by only 0.013 ms across imposed delays of 2.5–20.0 ms. The bias was frequency dependent, decreasing in magnitude from approximately 1.24 ms at 40 Hz to approximately 0.78 ms at 160 Hz under the 40 dB condition. No fallback detections or missed detections occurred. At 10 dB, the magnitude and variability of the bias increased, indicating reduced onset stability under lower-SNR conditions (Figure 3 and Supplementary Table S1).

### 3.3. Frequency-Dependent Shear-Wave Speed and KVFD Model-Dependent Fit Parameters

TOF-derived shear-wave speed increased with excitation frequency in all tissue conditions. In porcine liver, shear-wave speed increased from 2.437 m/s at 40 Hz to 3.866 m/s at 160 Hz. Chicken breast showed higher shear-wave speeds than porcine liver across the same frequency range. In the parallel muscle-fiber orientation, shear-wave speed increased from 4.953 m/s at 40 Hz to 7.127 m/s at 160 Hz, while in the perpendicular orientation, it increased from 4.814 m/s to 6.757 m/s. These results indicate frequency-dependent apparent shear-wave response across the tested tissue conditions.

The mean shear-wave speed across 40–160 Hz was lowest in porcine liver and higher in chicken breast. Porcine liver had a mean shear-wave speed of 3.145 m/s, whereas chicken breast measured parallel and perpendicular to the muscle fibers had mean shear-wave speeds of 6.133 m/s and 5.914 m/s, respectively. The corresponding KVFD elastic fit parameter *E*_0_ values were 9.55 ± 0.39 kPa for porcine liver, 19.36 ± 0.25 kPa for chicken breast parallel to the muscle fibers, and 17.02 ± 0.10 kPa for chicken breast perpendicular to the muscle fibers.

KVFD fitting showed good descriptive agreement with the measured frequency-dependent shear-wave-speed data. The mean sample-level R^2^ values were 0.990 for porcine liver, 0.984 for chicken breast measured parallel to the muscle fibers, and 0.983 for chicken breast measured perpendicular to the muscle fibers. The corresponding mean sample-level RMSE values were 0.0473, 0.095, and 0.0809 m/s, respectively. The mean apparent shear-wave speed, KVFD model-dependent fit parameters, and goodness-of-fit metrics are summarized in Table 2.

### 3.4. Direction-Dependent Shear-Wave Responses in Chicken Breast Muscles

**Figure 4:**
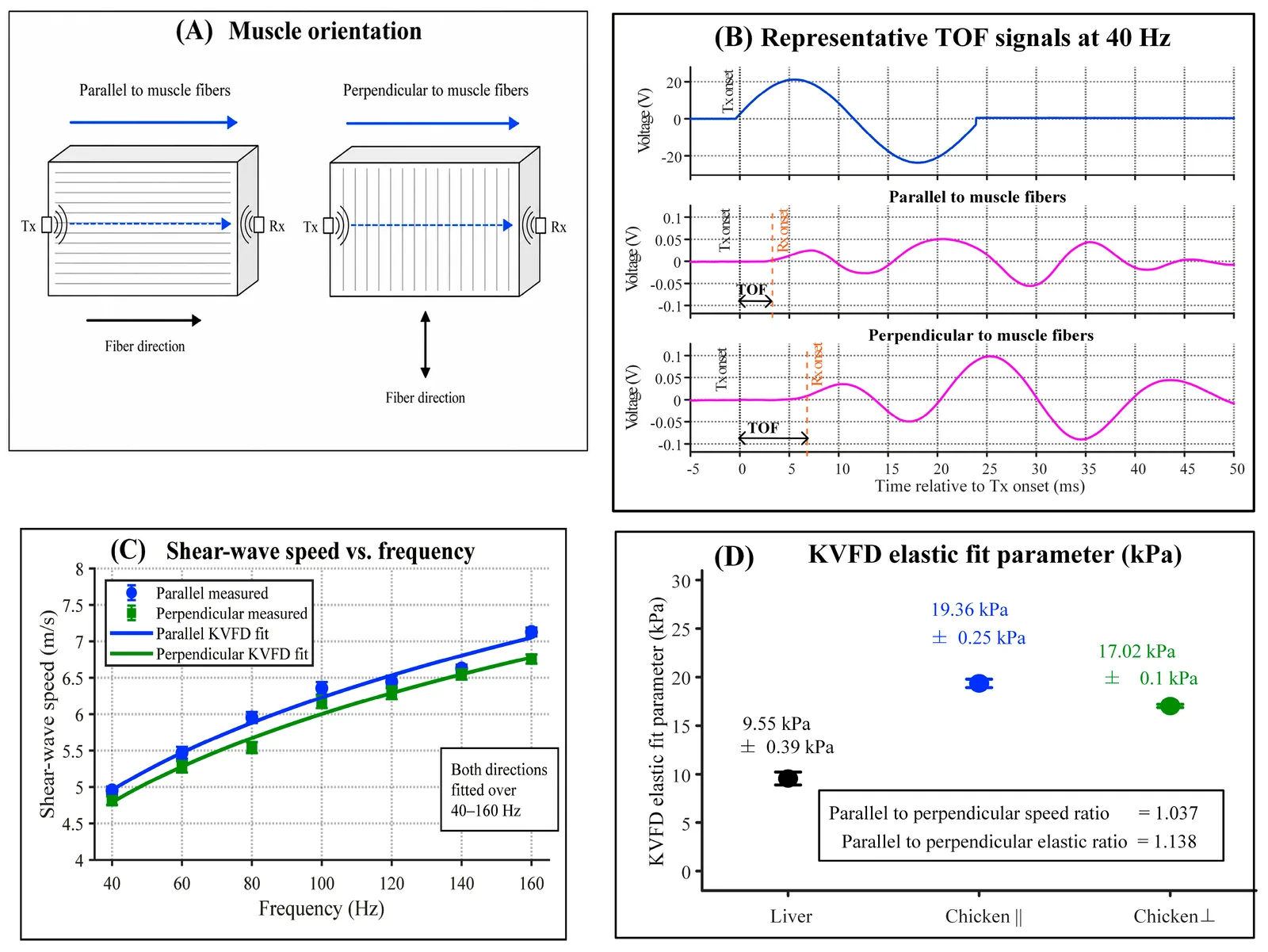
Direction-dependent shear-wave responses in chicken breast muscle measured using the TOF device. (**A**) Schematic illustration of the measurement orientations relative to the muscle-fiber direction. (**B**) Representative TOF signals at 40 Hz for chicken breast measured parallel and perpendicular to the muscle fibers. (**C**) Frequency-dependent shear-wave speed from 40 to 160 Hz with KVFD model fits for both orientations. (**D**) KVFD elastic fit parameter *E*_0_ for porcine liver, chicken breast parallel, and chicken breast perpendicular measurements.

Direction-dependent shear-wave responses in chicken breast muscle are shown in Figure 4. The measurement configuration defined two propagation directions relative to the visible muscle fibers: parallel and perpendicular (Figure 4A). Representative TOF signals at 40 Hz confirmed that received shear-wave responses were detectable in both orientations (Figure 4B).

Across the tested frequency range, shear-wave-speed was consistently higher in the parallel orientation than in the perpendicular orientation (Figure 4C). In the parallel orientation, shear-wave speed increased from 4.953 ± 0.055 m/s at 40 Hz to 7.127 ± 0.060 m/s at 160 Hz. In the perpendicular orientation, shear-wave speed increased from 4.814 ± 0.055 m/s to 6.757 ± 0.055 m/s over the same frequency range. Frequency-specific shear-wave speed values for both muscle orientations, with porcine liver included as a soft-tissue reference, are provided in Appendix A Table A1.

The direction-dependent summary is presented in Table 3. The mean apparent shear-wave speed across 40–160 Hz was 6.133 m/s in the parallel orientation and 5.914 m/s in the perpendicular orientation, corresponding to a parallel-to-perpendicular speed ratio of 1.037. The mean apparent shear-wave speed in the perpendicular direction was 3.56% lower than that in the parallel direction. Given the small number of paired biological samples (*n* = 3), this directional difference is presented descriptively, and no population-level statistical inference was performed.

KVFD elastic fit parameter, *E*_0_, showed the same directional pattern as the apparent shear-wave speed (Figure 4D; Table 3). The mean *E*_0_ value was 19.36 ± 0.25 kPa for chicken breast measured with propagation parallel to the muscle fibers and 17.02 ± 0.1 kPa for the perpendicular orientation. The perpendicular orientation therefore showed a 2.34 kPa lower *E*_0_ value, corresponding to a 12.09% reduction relative to the parallel orientation. The parallel-to-perpendicular *E*_0_ ratio was 1.138. These results indicate that both the onset-based apparent shear-wave speed and the KVFD elastic fit parameter exhibited a direction-dependent pattern, with higher values in the parallel orientation.

### 3.5. Signal-Quality Performance Across Tissue Type and Muscle Orientation

**Figure 5:**
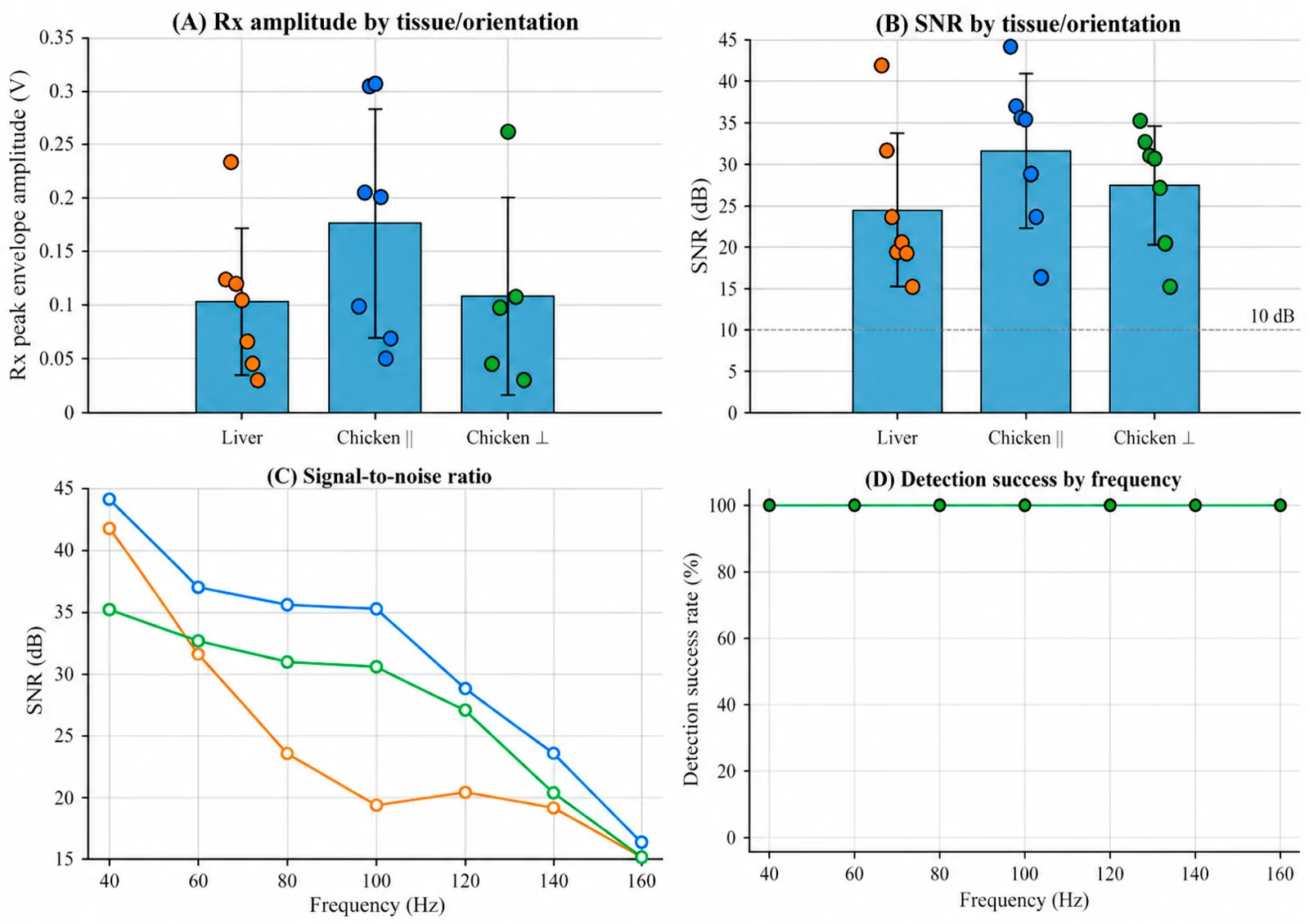
Signal-quality performance of TOF measurements in porcine liver and chicken breast measured along different muscle-fiber orientations. (**A**) Received peak envelope amplitude for porcine liver, chicken breast parallel, and chicken breast perpendicular measurements. Individual colored dots represent measurements obtained at the seven excitation frequencies (40–160 Hz), while bars and errors represent the group mean and variability, respectively (**B**) SNR for each tissue/orientation group. The dashed horizontal line indicates the 10 dB reference level used for visual interpretation. (**C**) Frequency-dependent SNR from 40 to 160 Hz. (**D**) TOF detection success rate across the tested frequencies. Porcine liver, chicken breast parallel, and chicken breast perpendicular measurements all achieved a 100% detection success rate at every tested frequency; therefore, the three series are completely superimposed.

Signal-quality performance was evaluated using received peak envelope amplitude, SNR, and TOF detection success rate across porcine liver, chicken breast parallel to the muscle fibers, and chicken breast perpendicular to the muscle fibers. The signal-quality results are shown in Figure 5 and summarized in Table 4.

The received peak envelope amplitude was comparable across the three tissue conditions, although chicken breast showed a higher mean received amplitude than porcine liver (Figure 5A). Porcine liver had a mean received peak envelope amplitude of 0.103 ± 0.068 V. Chicken breast measured parallel and perpendicular to the muscle fibers had mean amplitudes of 0.176 ± 0.106 V and 0.1086 ± 0.092 V, respectively. The larger standard deviation in the perpendicular orientation indicated greater variability in received signal amplitude across frequencies.

SNR remained above the 10 dB visual reference level for all tissue conditions across the tested frequency range (Figure 5B,C). Chicken breast measured parallel to the muscle fibers showed the highest mean SNR, at 31.52 ± 9.29 dB. Chicken breast measured perpendicular to the muscle fibers had a mean SNR of 27.44 ± 7.16 dB, while porcine liver had a mean SNR of 24.47 ± 9.18 dB. Across the tested frequencies, SNR generally decreased as excitation frequency increased, particularly in porcine liver and chicken breast measured perpendicular to the muscle fibers (Figure 5C).

Valid TOF detection was achieved at all tested excitation frequencies for all tissue conditions (Figure 5D). Porcine liver, chicken breast parallel, and chicken breast perpendicular each showed a detection success rate of 100% across 40–160 Hz. These results show that the TOF device produced detectable received responses with signal quality adequate for TOF estimation in porcine liver and both chicken breast measurement orientations.

### 3.6. Preliminary Descriptive Comparison with UD3000 SWE

A preliminary descriptive comparison between the KVFD elastic fit parameter, *E*_0_, and the stiffness values reported by the UD3000 SWE system is summarized in Table 5. The comparison was performed for porcine liver and chicken breast measured with propagation parallel to the muscle fibers. Because *E*_0_ was obtained by fitting the KVFD model to onset-based apparent propagation-speed data, it was treated as a model-dependent fit parameter and was not assumed to be mechanically equivalent to the stiffness reported by the UD3000.

For porcine liver, the UD3000 reported stiffness was 8.00 ± 0.54 kPa, whereas the KVFD elastic fit parameter *E*_0_ was 9.55 ± 0.39 kPa. The numerical difference was 1.55 kPa, corresponding to a symmetric percentage difference of 17.7%. For chicken breast measured parallel to the muscle fibers, the UD3000 reported stiffness was 22.36 ± 0.57 kPa, whereas *E*_0_ was 19.36 ± 0.25 kPa. The numerical difference was 3.00 kPa, corresponding to a symmetric percentage difference of 14.4%.

The two approaches produced the same relative tissue ranking, with higher numerical values for chicken breast than for porcine liver, and the reported values were of a similar order of magnitude. However, because the two quantities arise from different measurement principles and no predefined equivalence or agreement criterion was applied, the numerical differences and symmetric percentage differences were interpreted descriptively and do not demonstrate quantitative agreement, validation, or interchangeability between the two systems.

## 4. Discussion

### 4.1. Principal Findings and Scientific Interpretation

This study evaluated whether a custom shear-wave TOF device could detect frequency- and direction-dependent shear-wave responses in ex vivo biological tissues. The device detected received responses in porcine liver and chicken breast muscle, quantified frequency-dependent apparent shear-wave propagation speed, identified direction-dependent responses in chicken breast, and provided model-dependent KVFD fit parameters for describing the observed frequency dependence. The representative TOF measurement showed that the cumulative-energy algorithm identified the Rx onset after the transmitted excitation without manual peak selection (Figure 2). Across all tested tissue conditions, valid TOF detection was achieved at excitation frequencies ranging from 40 to 160 Hz, supporting the method’s feasibility under the ex vivo conditions tested (Figure 5D; Table 4).

The increase in TOF-derived apparent shear-wave speed with excitation frequency indicates a frequency-dependent apparent propagation response in the tested tissues (Table 2). Because the measured quantity was an onset-based apparent propagation-speed estimate rather than a direct phase-velocity measurement, the frequency-dependent apparent speeds were fitted using the KVFD relation only as a descriptive model of the observed frequency dependence. Accordingly, the fitted E0, η, and α values were treated as model-dependent KVFD fit parameters rather than as direct estimates of constitutive material properties. The observed increase in apparent shear-wave speed with excitation frequency is mechanically consistent with viscoelastic behavior, in which elastic storage and viscous dissipation contribute to frequency-dependent shear-wave propagation [12,28,29]. The KVFD model reproduced the measured frequency-dependent trend and provided a compact mathematical description of the data. The corresponding goodness-of-fit metrics (Table 2) describe how well the model represented the measured onset-based apparent speeds but do not establish the physical equivalence of those speeds to phase velocity or independently validate the fitted parameters as material constants.

The TOF measurements showed tissue-dependent apparent mechanical responses. Porcine liver exhibited lower apparent shear-wave speed and lower KVFD elastic fit parameter, *E*_0_, than chicken breast muscle (Table 2). This difference is unlikely to arise primarily from density. Under the simplified relation $c_{s}=\sqrt{G/\rho }$ where G is the shear modulus and *ρ* is the density, an apparent shear-wave speed in chicken breast approximately twice that in porcine liver would require the chicken breast density to be approximately one quarter of the liver density if the shear modulus were unchanged, which is not physically plausible for soft biological tissues. A common density of 1000 kg/m^3^ was assumed for all samples in the KVFD analysis (Section 2.7); therefore, tissue-specific density differences were not represented in the model-derived parameters. The higher apparent speed in chicken breasts is more plausibly associated with differences in its effective mechanical response and organized fiber structure compared with soft liver parenchyma. However, because only three porcine liver samples and three chicken breast samples were examined, these findings should be interpreted as evidence of tissue-dependent sensitivity under the present ex vivo conditions rather than as a population-level comparison of liver and muscle mechanical properties.

A further finding was the detection of direction-dependent shear-wave response in chicken breast muscle. Apparent shear-wave speed was consistently higher when propagation was oriented parallel rather than perpendicular to the visible muscle fibers across the tested frequency range (Figure 4C; Supplementary Table S1). The KVFD elastic fit parameter, *E*_0_, showed the same directional pattern, with a parallel-to-perpendicular *E*_0_ ratio of 1.138 (Figure 4D; Table 3). This difference indicates an additional contribution from propagation direction relative to the organized muscle fibers and is consistent with previous elastography studies showing that shear-wave propagation in skeletal muscle depends on fiber alignment [19,30]. Nevertheless, the present findings are interpreted as direction-dependent TOF responses under the tested experimental configuration rather than as definitive evidence of intrinsic muscle anisotropy, because independent mechanical anisotropy testing and quantitative control of interface compression were not performed.

The signal-quality analysis further supports the feasibility of the measurement approach. Received peak envelope amplitude and SNR were measurable in all tissue conditions, and all groups maintained SNR above the 10 dB visual reference level across the tested frequency range (Figure 5A–C; Table 4). Chicken breast measured parallel to the muscle fibers produced the highest mean SNR, whereas porcine liver and chicken breast perpendicular measurements showed lower SNR values and stronger frequency-related decline. This trend is important because signal attenuation, coupling, and direction-dependent tissue structure can influence the reliability of TOF detection at higher frequencies. Nevertheless, the 100% detection success rate across 40–160 Hz indicates that the selected frequency range was appropriate for the present ex vivo tissue measurements (Figure 5D; Table 4).

The preliminary comparison with UD3000 SWE provided descriptive context for the KVFD elastic fit parameter, *E*_0_. The two approaches produced the same relative tissue ranking, with higher numerical values for chicken breast measured parallel to the muscle fibers than for porcine liver, and the values were of a similar order of magnitude (Table 5). However, *E*_0_ and the UD3000-reported stiffness were not assumed to represent mechanically equivalent quantities. The TOF device and UD3000 SWE differ in excitation mechanism, frequency content, sensing approach, ROI definition, and reconstruction assumptions; therefore, the comparison was interpreted only descriptively and does not establish quantitative agreement, validation, equivalence, or interchangeability between the two systems [31,32,33]. The methodological contribution of the TOF approach lies in its controlled, frequency-specific estimation of apparent shear-wave propagation speed using a known propagation distance and explicit onset-based TOF detection.

Overall, the findings support the feasibility of the TOF device as a controlled research platform for investigating frequency and direction-dependent shear-wave responses in averaged ex vivo signals. The approach combined low-frequency mechanical excitation, Tx–Rx onset-delay measurement, cumulative-energy-onset detection, frequency-dependent apparent shear-wave-speed analysis, and descriptive KVFD model fitting. The results extend the previous phantom-based application of the TOF approach toward ex vivo biological tissue, while also demonstrating the need for further calibration of the electromechanical delay, improved uncertainty and coupling control, larger biological sample sets and independent mechanical reference testing before the fitted KVFD parameters can be interpreted quantitatively as constitutive tissue properties or claims of absolute stiffness accuracy, intrinsic muscle anisotropy, or clinical interchangeability can be made.

### 4.2. Limitations and Future Work

This study has several limitations. The experiments were performed using three porcine liver samples and three chicken breast samples; therefore, the findings should be interpreted as an ex vivo feasibility assessment rather than a population-level comparison of tissue mechanical properties. Ex vivo tissue properties may also be affected by post-procurement handling, hydration state, temperature, and time after collection. Although the samples were stored in PBS and measured approximately 5 h after procurement, these conditions do not fully reproduce the in vivo environment.

The KVFD parameters were obtained by fitting the model to frequency-dependent onset-based apparent shear-wave-speed data. Because the measured propagation speed was not a direct phase-velocity measurement, the fitted *E*_0_, *η*, and *α* values were treated strictly as model-dependent fit parameters rather than as direct estimates of Young’s modulus, viscosity, or other constitutive tissue properties. No rheometric, tensile, compression, or independent mechanical reference measurements were performed; therefore, physical interpretation of the fitted KVFD parameters as material properties and absolute mechanical validation cannot be established from the present data.

Validation using synthetic signals with known arrival times showed that the combined Rx preprocessing and cumulative-energy onset-detection procedure identified the imposed arrival systematically early, with a condition-specific mean bias of approximately 0.75–1.37 ms at SNRs of 20–40 dB. Although the bias was largely independent of the imposed delay at a fixed frequency and SNR, its magnitude varied with excitation frequency and may therefore affect both the absolute apparent propagation speeds and their observed frequency-dependent trends. Frequency-specific bias-adjusted apparent speeds were examined as a sensitivity analysis (Supplementary Table S1). The adjustment reduced the absolute apparent speeds but preserved the principal comparative findings, including the frequency-dependent apparent response, the parallel-to-perpendicular difference and the separation between porcine liver and chicken breast.

A direct numerical correction was not applied because the bias was characterized using idealized, non-dispersive bursts and may not transfer directly to experimental tissue signals affected by attenuation, system response, coupling, and frequency-dependent propagation characteristics. The KVFD model was not refitted using the adjusted apparent shear-wave speeds because this would propagate uncertainty from a simulation-derived correction into the jointly estimated model parameters. Accordingly, no bias-adjusted KVFD fit parameters are reported.

Although the Tx–Rx propagation distance was remeasured after each sample reorientation and the starting orientation was alternated across samples, contact force and tissue compression at the actuator and receiver interfaces were not quantitatively controlled. Residual differences in coupling, local compression, or mechanical loading may therefore have contributed to the observed parallel-to-perpendicular differences. In addition, onset timing was limited to the native temporal sampling interval of approximately 0.05 ms.

Accordingly, the findings are interpreted as direction-dependent TOF responses under the tested experimental configuration rather than as definitive evidence of intrinsic muscle anisotropy. The resolution-based uncertainty estimate did not account for systematic onset-timing bias, residual electromechanical system delay, coupling or tissue-compression effects; therefore, the reported shear-wave speeds are interpreted as onset-based apparent propagation speeds, while the KVFD outputs are interpreted as model-dependent fit parameters.

The preliminary UD3000 comparison was descriptive and limited to porcine liver and chicken breast measured parallel to the muscle fibers. The KVFD elastic fit parameter *E*_0_ and the UD3000-reported stiffness were not assumed to represent mechanically equivalent quantities. The TOF device and UD3000 SWE differ in excitation mechanism, frequency content, sensing approach, ROI definition, and reconstruction assumptions; consequently, the comparison provides only descriptive information on relative tissue ranking and order of magnitude and does not establish quantitative agreement, equivalence, validation, or interchangeability between the systems (Table 5). The tested frequency range was also limited to 40–160 Hz, and the results may not generalize to higher frequencies, other tissue types, diseased tissues, or in vivo measurement conditions.

Future work should include multiple independent biological tissue samples, controlled temperature and hydration monitoring, improved control of tissue coupling and contact pressure, direct comparison with mechanical reference testing, and larger paired comparisons with clinical elastography systems. Future studies should also investigate the relationship between onset-based apparent propagation speed and conventional phase velocity through appropriate theoretical, numerical, or experimental approaches before the fitted KVFD parameters are interpreted as constitutive tissue properties. These steps will be necessary to determine repeatability, biological variability, the accuracy and physical interpretation of the TOF-derived apparent propagation measurements, and the broader applicability of the TOF device for frequency-dependent tissue characterization.

## 5. Conclusions

This study demonstrated that a custom shear-wave TOF device could detect frequency-dependent responses in filtered, 50-shot ensemble-averaged signals from ex vivo porcine liver and chicken breast over 40–160 Hz, with all 63 sample–frequency measurements satisfying the predefined primary-detection criteria. The measured apparent shear-wave propagation speeds differed between tissue conditions, and chicken breast exhibited direction-dependent responses when the propagation path was oriented parallel and perpendicular to the visible muscle fibers. KVFD fitting provided model-dependent fit parameters for descriptively representing the observed frequency-dependent onset-based apparent propagation-speed data. The preliminary comparison with UD3000 SWE showed the same relative tissue ranking, with values of a similar order of magnitude; however, the KVFD elastic fit parameter *E*_0_ and the UD3000-reported stiffness were not assumed to be mechanically equivalent, and the comparison was descriptive rather than a validation or agreement analysis.

These findings support the feasibility of the device as a controlled research platform for investigating ex vivo shear-wave propagation. However, the reported apparent shear-wave speeds should not be interpreted as direct phase-velocity measurements, and the fitted KVFD parameters should not be interpreted as validated constitutive tissue properties. The parallel-to-perpendicular differences do not establish intrinsic muscle anisotropy. Further calibration of the frequency-dependent electromechanical delay, improved characterization of measurement uncertainty and control of tissue coupling, retention of individual excitation responses, larger biological sample sets, and independent mechanical reference testing are required before claims of absolute mechanical-property measurement accuracy, muscle anisotropy, or clinical interchangeability can be made.

## Figures and Tables

**Table 1:** Tissue samples and experimental measurement conditions.

Tissue Sample	Biological Tissue Class	Sample Number	Measurement Orientation	Tx–Rx Distance (mm)
Porcine Liver	Soft parenchymal tissue	3	No directional alignment	22.42
Chicken breast	Skeletal muscle tissue	3	Parallel to muscle fibers	23.52
Chicken breast	Skeletal muscle tissue	3	Perpendicular to muscle fibers	23.52

**Table 2:** TOF-derived apparent SWS, KVFD model-dependent fit parameters, and model-fitting metrics for ex vivo porcine liver and chicken breast.

Tissue/Orientation	Mean Apparent SWS Across 40–160 Hz (m/s)	KVFD Elastic Fit Parameter, *E*_0_ (kPa)	KVFD Viscosity- Related Parameter, *η* (Pa·s^α^)	KVFD Fractional Order, *α*	Mean Sample-Level *R*^2^	Mean Sample-Level RMSE (m/s)
Porcine liver	3.145	9.55 ± 0.39	106 ± 3.06	0.81 ± 0.007	0.99	0.0473
Chicken breast parallel	6.133	19.36 ± 0.25	2265 ± 23.24	0.57 ± 0.003	0.984	0.095
Chicken breast perpendicular	5.914	17.02 ± 0.10	2253.33 ± 18.56	0.57 ± 0.0063	0.983	0.0809

**Table 3:** Direction-dependent apparent SWS and KVFD elastic fit parameter *E*_0_ in chicken breast muscle.

Parameter	Value
Mean SWS $c_{∥}\left(f\right),$ across 40–160 Hz	6.133 m/s
Mean SWS $c_{⊥}\left(f\right),$ across 40–160 Hz	5.914 m/s
Parallel to perpendicular SWS ratio $\left(\bar{c_{∥}}/\bar{c_{⊥}}\right)$	1.037
Mean SWS difference	3.56% lower in the perpendicular direction
KVFD elastic fit parameter $\left(E_{0∥}\right)$	19.36 ± 0.25 kPa
KVFD elastic fit parameter $\left(E_{0⊥}\right)$	17.02 ± 0.1 kPa
*E*_0_ difference, $\left(E_{0∥}-E_{0⊥}\right)$	2.34 kPa
*E*_0_ reduction in the perpendicular direction	12.09%
Parallel to perpendicular *E*_0_ ratio, $\left(E_{0∥}/E_{0⊥}\right)$	1.138

**Table 4:** Signal-quality summary for TOF measurements in porcine liver and chicken breast.

Tissue/Orientation	Received Peak Envelope Amplitude (V) Mean ± SD	SNR (dB) Mean ± SD	Primary Detections, n/N (%)
Porcine liver	0.103 ± 0.068	24.47 ± 9.18	21/21 (100%)
Chicken breast parallel to fibers	0.176 ± 0.106	31.52 ± 9.29	21/21 (100%)
Chicken breast perpendicular to fibers	0.1086 ± 0.092	27.44 ± 7.16	21/21 (100%)

**Table 5:** Preliminary descriptive comparison of the KVFD elastic fit parameter *E*_0_ and UD3000 reported stiffness.

Tissue	UD3000 Reported Stiffness (kPa), Mean ± SD	KVFD Elastic Fit Parameter, *E*_0_ (kPa)	Numerical Difference (kPa)	Symmetric Percentage Difference (%)
Porcine liver	8.00 ± 0.54	9.55 ± 0.39	1.55	17.7
Chicken breast, parallel to fibers	22.36 ± 0.57	19.36 ± 0.25	3	14.4

## Data Availability

All relevant data are within the manuscript.

## Supplementary Materials

The following supporting information can be downloaded at: https://www.mdpi.com/article/10.3390/bioengineering13090959/s1, Figure S1: Adaptive FIR filter responses at all tested excitation frequencies; Table S1: Frequency-specific onset-detection bias and sensitivity analysis of apparent propagation velocities; Note S1: MATLAB implementation of the frequency-adaptive FIR filter.

## Appendix A

**Table A1:** Frequency-dependent apparent SWS in porcine liver and chicken breast measured parallel and perpendicular to the muscle fibers.

Frequency (Hz)	Porcine Liver SWS (m/s) Mean ± SE	Chicken Breast Parallel SWS (m/s) Mean ± SE	Chicken Breast Perpendicular SWS (m/s) Mean ± SE	Difference Between Chicken Orientations (%)	Parallel to Perpendicular Apparent-SWS Ratio
40	2.437 ± 0.0374	4.953 ± 0.055	4.814 ± 0.058	2.806	1.029
60	2.685 ± 0.0544	5.470 ± 0.080	5.279 ± 0.078	3.492	1.036
80	2.892 ± 0.0510	5.954 ± 0.075	5.541 ± 0.075	6.936	1.075
100	3.092 ± 0.0578	6.357 ± 0.085	6.169 ± 0.087	2.957	1.03
120	3.397 ± 0.0568	6.444 ± 0.085	6.294 ± 0.088	2.328	1.024
140	3.646 ± 0.0374	6.625 ± 0.060	6.546 ± 0.070	1.193	1.012
160	3.866 ± 0.0408	7.127 ± 0.060	6.757 ± 0.064	5.191	1.055
