# Supplementary material for: Frequency and Direction-Dependent Shear-Wave Responses in Ex Vivo Tissues Measured by a Time-of-Flight Device
Source: Bioengineering (Basel). 2026 Aug 23;13(9):959. doi: 10.3390/bioengineering13090959 (PMC13603093; doi:10.3390/bioengineering13090959)

Adaptive FIR filter responses at all tested excitation frequencies (order = 50, f_s = 20.0 kSa/s)

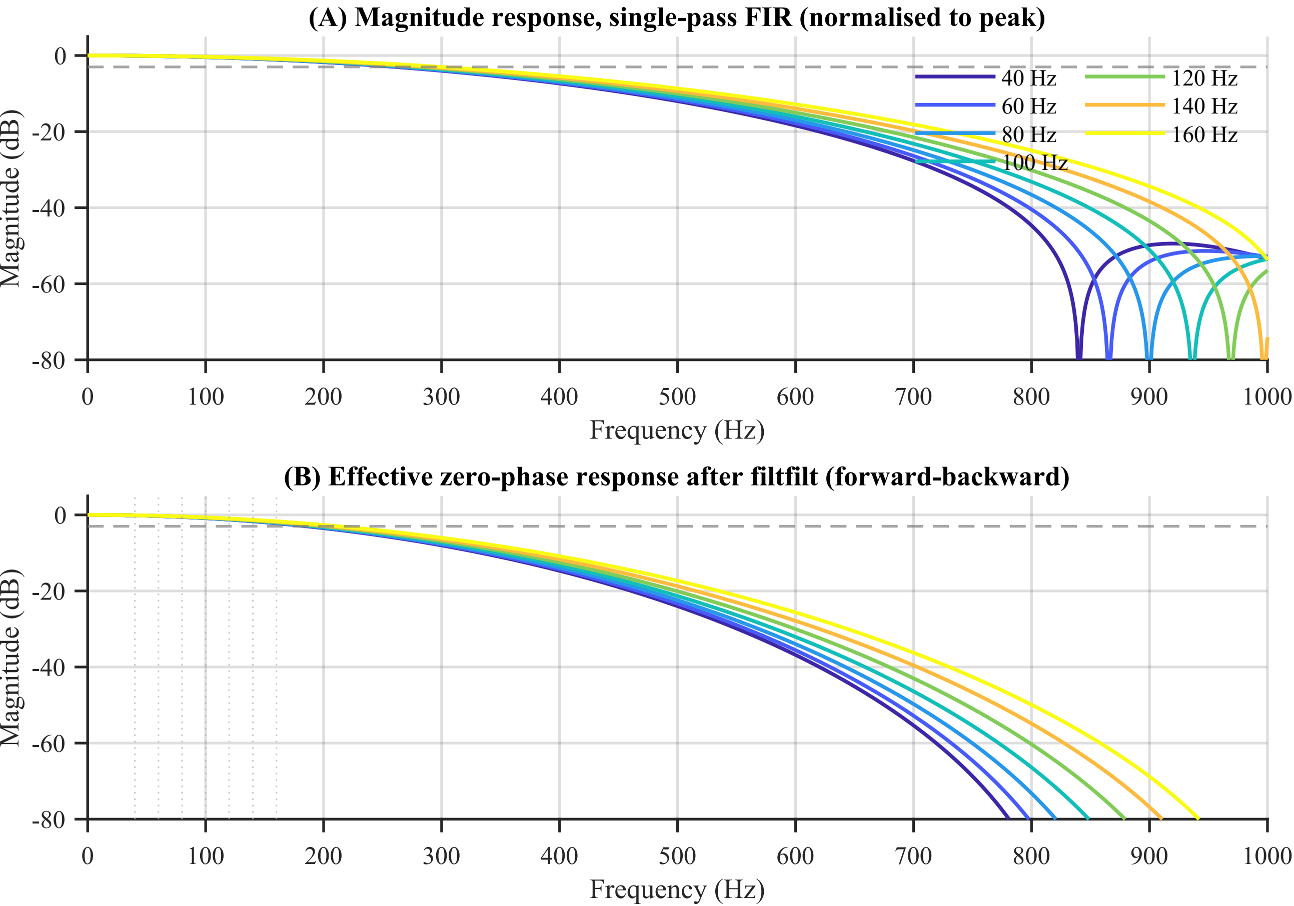

Supplement: Supplementary file 1 [file bioengineering-13-00959-s001.zip › Supplementary Materials/Figure_S1.pdf]
