# Supplementary material for: Frequency and Direction-Dependent Shear-Wave Responses in Ex Vivo Tissues Measured by a Time-of-Flight Device
Source: Bioengineering (Basel). 2026 Aug 23;13(9):959. doi: 10.3390/bioengineering13090959 (PMC13603093; doi:10.3390/bioengineering13090959)
Supplement: Supplementary file 1 [file bioengineering-13-00959-s001.zip › Supplementary Materials/Supplementary Note S1.pdf]

Supplementary NoteS1. MATLAB implementation of the frequency-adaptive FIR filter

The filter was designed and applied using the following MATLAB commands:

```
bw = max(20, round(2 * tx_freq / tx_cycles));  % “tx_freq” is the excitation frequency
low_cut  = max(1, tx_freq - bw/2);                  % fs = Sampling frequency
high_cut = min(fs/2 - 1, tx_freq + bw/2);
Wn = [low_cut, high_cut] / (fs/2);
bp_fir = fir1( ...
    fir_order, ...
    Wn, ...
    'bandpass', ...
    hamming(fir_order + 1));
ch1_bp = filtfilt(bp_fir, 1, ch1_med);
ch2_bp = filtfilt(bp_fir, 1, ch2_med);
```
