# Supplementary material for: Frequency and Direction-Dependent Shear-Wave Responses in Ex Vivo Tissues Measured by a Time-of-Flight Device
Source: Bioengineering (Basel). 2026 Aug 23;13(9):959. doi: 10.3390/bioengineering13090959 (PMC13603093; doi:10.3390/bioengineering13090959)
Supplement: Supplementary file 1 [file bioengineering-13-00959-s001.zip › Supplementary Materials/Supplementary Table S1.pdf]

Table S1. Frequency-specific onset-detection bias and sensitivity analysis of apparent propagation velocities

Panel A. Frequency-specific onset bias and apparent propagation velocities

Frequency (Hz)	Mean onset bias (ms)	Liver reported (m/s)	Liver adjusted (m/s)	Chicken parallel reported (m/s)	Chicken parallel adjusted (m/s)	Chicken perpendicular reported (m/s)	Chicken perpendicular adjusted (m/s)
40	−1.240	2.437	2.148	4.953	3.927	4.814	3.84
60	−1.029	2.685	2.39	5.47	4.414	5.279	4.288
80	−0.883	2.892	2.596	5.954	4.866	5.541	4.587
100	−0.827	3.092	2.776	6.357	5.196	6.169	5.07
120	−0.784	3.397	3.036	6.444	5.305	6.294	5.203
140	−0.782	3.646	3.235	6.625	5.429	6.546	5.376
160	−0.781	3.866	3.407	7.127	5.763	6.757	5.519

Panel B. Sensitivity of the principal comparative metrics

Comparative metric	Reported value	Bias-adjusted value
Liver dispersion ratio, c_{160}/c_{40}	1.586	1.587
Chicken parallel dispersion ratio, c_{160}/c_{40}	1.439	1.467
Chicken perpendicular dispersion ratio, c_{160}/c_{40}	1.404	1.437
Mean parallel-to-perpendicular speed ratio	1.0372	1.0302
Mean chicken-parallel-to-liver speed ratio	1.963	1.791

Note: Onset bias was defined as detected onset minus imposed true onset; negative values therefore indicate early detection. Frequency-specific bias values represent the mean errors obtained using synthetic signals at SNRs of at least 20 dB, with 20 noise realizations at each of six imposed delays spanning 2.5–20 ms. Bias-adjusted velocities are presented only to evaluate the robustness of the comparative findings and were not substituted into the primary analysis. The KVFD model was not refitted using the adjusted velocity curves; therefore, no bias-adjusted stiffness parameters are reported.
